# Shaping next decade of systemic therapy for head and neck squamous cell carcinoma: where do we go next?

**DOI:** 10.3389/fonc.2026.1799378

**Published:** 2026-03-26

**Authors:** Michael Saerens, Tijl Vermassen, Jenas Stevenheydens, Willem Lybaert

**Affiliations:** 1 Medical Oncology, University Hospital Ghent, Ghent, Belgium; 2 Vlaamse Werkgroep Hoofd-HalsTumoren (VWHHT), Ghent, Belgium; 3 Vlaams Instituut Biotechnologie , Center for Medical Biotechnology, Ghent, Belgium; 4 Cancer Research Institute Ghent, Ghent University, Ghent, Belgium; 5 Clinical Trials Office, VITAZ, Sint-Niklaas, Belgium; 6 Department of Medical Oncology, VITAZ, Sint-Niklaas, Belgium; 7 Universitair Ziekenhuis Antwerpen, Edegem, Belgium

**Keywords:** bispecific antibodies, head and neck squamous cell carcinoma, HNSCC, immune checkpoint inhibitor, immunotherapy, oncolytic vaccines, therapeutic vaccine

## Abstract

Outcomes of patients with relapsed or metastatic head and neck squamous cell carcinoma (R/M HNSCC) remain poor, despite the widespread incorporation of immune checkpoint inhibitors into contemporary treatment algorithms. Although programmed death-1 (PD-1) blockade with pembrolizumab, administered either alone or in combination with chemotherapy, has become the cornerstone of first line therapy for patients with PD-L1 positive disease, durable clinical benefit is achieved in only a minority of cases. This narrative review explores the most promising systemic treatment strategies expected to shape the management of R/M HNSCC over the coming decade, including bispecific antibodies, HPV directed therapeutic vaccines, antibody–drug conjugates, and other emerging modalities. We discuss their underlying mechanisms of action, review clinical data from early phase studies, and highlight ongoing phase III trials with the potential to redefine future treatment paradigms in R/M HNSCC.

## 1 Introduction

The therapeutic landscape of relapsed or metastatic head and neck squamous cell carcinoma (R/M HNSCC) has evolved substantially since the introduction of anti−programmed death-1 (anti-PD1) targeting agents into routine clinical practice (1–3). Despite these advances, overall survival (OS) remains poor for a considerable proportion of patients, underscoring a persistent and substantial unmet clinical need. In particular, there is an urgent requirement to enhance current immune checkpoint inhibitor (ICI)−based strategies in the first−line setting and to expand effective treatment options for patients who experience disease progression following immunotherapy. Advances in translational research has unraveled primary and secondary resistance mechanisms to ICIs, opening frontiers for new treatment algorithms. In parallel, a more refined understanding of the heterogenous biology of HNSCC, in particular human papillomavirus (HPV)–associated tumors, provide a strong biological rationale for the development for a more individualized and mechanistic approach. In this context, the present review focuses on the next generation of systemic agents in HNSCC, with particular emphasis on bispecific antibodies and HPV−directed therapeutic vaccines, which currently represent the most promising therapeutic classes transitioning from encouraging phase I/II signals toward definitive phase III clinical evaluation.

## 2 Methods

A comprehensive literature search was conducted using Pubmed to identify relevant clinical and translational studies in R/M HNSCC. Following search terms were used: head and neck squamous cell carcinoma, HNSCC, head and neck cancer, relapsed, metastatic, R/M HNSCC, treatment. To ensure inclusion of recent, unpublished studies, abstracts from the American Society of Clinical Oncology (ASCO) and the European Society of Medical Oncology (ESMO) Annual Conference from 2023 to 2025 were screened for results of recently completed trials in R/M HNSCC.

Because the aim was to provide a forward-looking review, we prioritized randomized phase II and III clinical trials. Early phase trials were considered if a randomized phase 3 study was announced on clinicaltrials.gov (accessed on Jan 15 2026). Data extraction included: mechanism of action, study design and patient population, objective response rate, progression-free survival, overall survival, status of ongoing clinical development. Given the narrative design of this review, no formal quality assessment or meta-analysis was performed.

## 3 Current treatment algorithm in R/M HNSCC

The pivotal KEYNOTE−048 trial fundamentally reshaped the first−line treatment paradigm for R/M HNSCC by introducing biomarker−driven therapeutic decision−making based on the PD−L1 Combined Positivity Score (CPS) (4). In this large phase III study, 882 patients were randomized in a 1:1:1 ratio to receive pembrolizumab monotherapy, pembrolizumab combined with platinum−based chemotherapy (cisplatin or carboplatin plus 5−fluorouracil), or the then standard−of−care EXTREME regimen consisting of platinum, 5−fluorouracil, and cetuximab. Among patients with a PD−L1 CPS ≥ 1, OS was significantly prolonged with both pembrolizumab monotherapy (HR 0.74, 95%CI 0.61-0.89) and pembrolizumab combined with chemotherapy (HR 0.64, 95%CI 0.53-0.78) compared with cetuximab−based treatment,. In contrast, no survival benefit was observed for either pembrolizumab−containing regimen in patients with PD−L1 CPS < 1 (1).

Although the addition of platinum−based chemotherapy to pembrolizumab resulted in higher objective response rates (ORRs) compared with pembrolizumab monotherapy, this benefit came at the cost of increased toxicity, and the KEYNOTE−048 trial was not powered to demonstrate a survival difference between the two pembrolizumab−based arms. In this context, alternative chemotherapy backbones have also been explored. The single−arm phase IV KEYNOTE−B10 trial reported an ORR of 49% with the combination of carboplatin, paclitaxel, and pembrolizumab, offering a clinically relevant option for patients requiring chemotherapy who are unsuitable for 5−fluorouracil (5). Nevertheless, for patients with PD−L1 CPS < 1 or for those with contraindications to immune checkpoint inhibition, cetuximab combined with chemotherapy remains the standard systemic treatment approach (6).

Importantly, this established treatment algorithm is likely to evolve after the introduction of ICIs in the locally advanced (LA) setting (7, 8). Indeed, the addition of neoadjuvant and adjuvant pembrolizumab in patients with resectable LA HNSCC significantly improved event−free survival (EFS) in patients with a PD-L1 CPS >1 (8). Similarly, the NIVOPOSTOP trial demonstrated that the addition of nivolumab to postoperative cisplatin−based radiotherapy improved disease−free survival in patients with high−risk, resected LA HNSCC, irrespective of PD−L1 CPS status (7). Albeit the improvement in EFS is significant, still >40% of patients will develop a recurrence or death within three years.

Data guiding the optimal management after receiving ICIs in the locally advanced setting are lacking and translational insights into mechanisms of resistance in this context remain limited. Retreatment with anti−PD−1 agents may be considered in carefully selected patients who experienced a prior benefit and maintained a treatment−free interval of at least six months, although the evidence supporting this strategy is sparse and largely extrapolated from other tumor types (9). Collectively, the expanding use of immune checkpoint inhibitors in earlier disease settings, together with the substantial failure rates observed in R/M HNSCC, highlight a growing unmet need for effective therapeutic strategies in patients with disease progression following anti−PD−1 exposure.

## 4 Attempts to overcome primary immunotherapy resistance: rational strategies, limited impact

**Figure 1:**
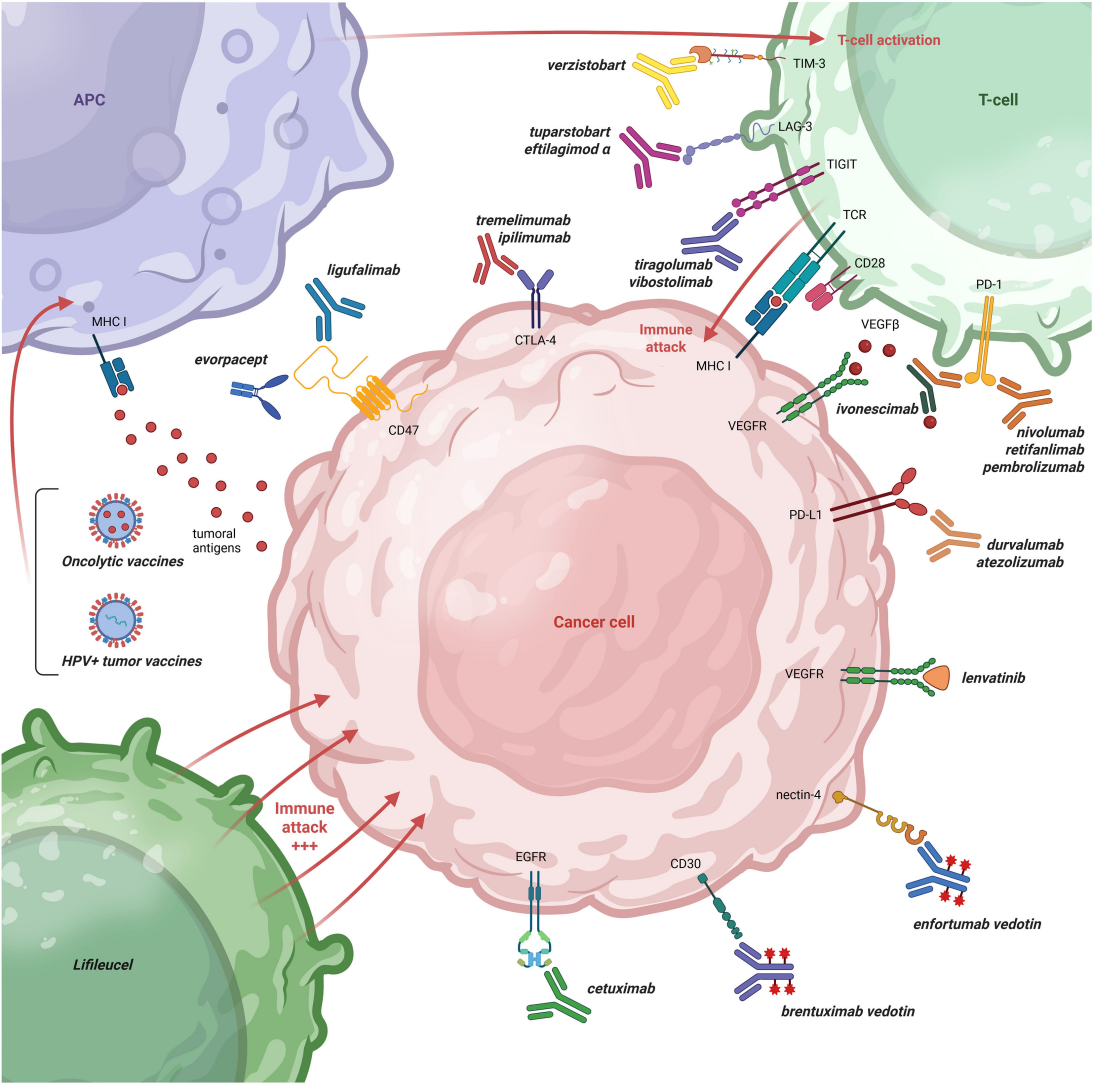
Overview of current and future targets in R/M HNSCC. The most commonly used type of immune checkpoint inhibitors (ICIs) are anti-PD1 (*nivolumab*, *pembrolizumab*, *retifanlimab*) and anti-PD-L1 (*atezolizumab*, *durvalumab*) monoclonal antibodies (moAbs). Overcoming resistance to anti-PD(L)1 based treatments has been explored by (1) co-targeting other immune checkpoints such as CTLA-4 moAbs (*ipilimumab*, *tremelimumab*); TIGIT (*tiragolumab*, *vibostolimab*), LAG-3 (*tuparstobart*, *eftilagimod α*), and TIM-3 (*verzistobart*) (2) inhibit immune-inhibitory signals, such as CD47 = ‘do not eat me’ signal (*ligufalimab*, *evorpacept*); (3) reducing hypoxia by adding anti-VEGF targeting agents (*lenvatinib, ivonescimab*); (4) targeting EGFR (*cetuximab, amivantamab, petosemtamab, ficerafusb alfa*); (5) use of ADC targeting tumoral surface markers such as nectin-4 (*enfortumab vedotin*) or CD30 (*brentuximab vedotin*); (6) enhancing the tumor-specific immune response via *oncolytic vaccines* (eg *TG4050*, *HPV^+^ therapeutic vaccines)* or *ex vivo* expanded tumor-directed T-cells (*lifileucel*). ADC, antibody-drug conjugate; APC, antigen presenting cell; CTLA-4, cytotoxic T-lymphocyte-associated protein 4; EGFR, epidermal growth factor receptor; HPV, human papillomavirus; ICIs, immune checkpoint inhibitors; LAG-3, lymphocyte-activation gene 3; MHC, major histocompatibility complex; PD-1, programmed death-1; PD-L1, programmed death-ligand 1; R/M HNSCC, recurrent/metastatic head and neck squamous cell carcinoma; TCR, T-cell receptor; TIGIT, T-cell immunoreceptor with immunoglobulin and ITIM domains; TIM-3, T-cell immunoglobulin and mucin-domain containing-3; VEGFR, vascular endothelial growth factor receptor; VEGFβ, vascular endothelial growth factor beta. Figure created in BioRender.

A thorough understanding of both intrinsic and adaptive resistance mechanisms to anti−PD−1 therapy is essential to meaningfully improve clinical outcomes in patients with R/M HNSCC. Mechanistically, resistance to immunotherapy in HNSCC is largely driven by a combination of immune evasion strategies and adaptive tumor−intrinsic signaling alterations (10, 11). These processes encompass the upregulation of immunosuppressive mediators within the tumor microenvironment (TME), defects in antigen processing and presentation, and activation of alternative growth factor signaling pathways, which can all be targeted specifically (**Figure 1**).

Multiple strategies have been explored to overcome immune resistance; however, to date, most have yielded disappointing clinical results (see **Table 1**). Numerous clinical trials have investigated dual immune checkpoint inhibition in this setting, similar to eg melanoma and renal cell cancer (21, 22). While the combination of anti−CTLA−4 and anti−PD−1 therapy can elicit a more pleiotropic immune response—characterized in part by increased T−cell receptor repertoire diversity—the addition of anti−CTLA−4 to anti−PD−(L)1 blockade in R/M HNSCC patients failed to improve OS in either the KESTREL trial (HR 1.05; 95% CI 0.80–1.39) or the CHECKMATE−651 study (HR 0.95; 97.9% CI 0.80–1.13; p = 0.495) (13, 14).

**Table 1:** Overview of randomized trials with immune checkpoint inhibitors in 1^st^ line R/M HNSCC.

Author, year	Intervention	Control	Population	ORR	mPFS	mOS
Harrington, 2022 (12)	pembrolizumab or pembrolizumab + chemotherapy	EXTREME	1L, all comers (N = 882)	19%* 37%*	3.2m* 5.1m*	12.3m* 13.7m*
Dzienis, 2024 (5)	pembrolizumab + carboplatin + paclitaxel	–	1L, all comers (N = 101)	49%	5.6m	13.1m
Psyrri,2023 (13)	durvalumab or durvalumab + tremelimumab	EXTREME	1L all comers (N = 825)	17% 21%	2.8m 2.8m	9.9m 10.7m
Haddad, 2023 (14)	ipilimumab + nivolumab	EXTREME	1L, all comers (N = 947)	24%	4.2m*	15.7m*
Kristensen, 2024 (15)	eftilagimod alpha + pembrolizumab	pembrolizumab	1L, PD-L1 CPS >1 (N = 138)	33%	NA	NA
Psyrri, 2025 (16)	atezolizumab + tiragolumab	atezolizumab	1L, PD-L1 TPS >5 (N = 119)	21%	4.1m	16.2m
Haddad, 2025 (17)	retifanlimab + tubarstobart +/- verzistobart	retifanlimab	1L, PD-L1 CPS >1 (N = 176)	30% 32%	5.8m 5.3m	NA
Licitra, 2024 (18)	pembrolizumab + lenvatinib	pembrolizumab	1L, PD-L1 CPS >1 (N = 511)	46%	6.2m	15.0m
Harrington, 2025(K.J. 19)	pembrolizumab + chemotherapy + evorpacept	pembrolizumab + chemo	1L, PD-L1 CPS >1 (N = 165)	37%	5.6m	15.5m
Keam, 2025 (20)	pembrolizumab + evorpacept	pembrolizumab	1L, PD-L1 CPS >1 (N = 181)	26%	3.6m	15.5m

*survival estimates in PD-L1 CPS >1 population.

EXTREME , cisplatin + 5FU + cetuximab.

R/M HNSCC, relapsed or metastatic head and neck squamous cell carcinoma; 1L, first line treatment; PD-L1 CPS, combined positivity score of PD-L1 expression; ORR, objective response rate; mPFS, median progression-free survival; mOS, median overall survival; NA, not available; NR, not reached.

Within this context, eftilagimod alpha (efti), a soluble LAG−3 protein targeting a subset of MHC class II molecules, has emerged as a novel immunomodulatory agent capable of enhancing antigen−presenting cell activation and CD8+ T−cell responses. In combination with pembrolizumab, efti achieved a numerically higher ORR of 32.8% compared with 26.7% for pembrolizumab monotherapy in patients with PD−L1 CPS ≥ 1, although survival outcomes for this cohort have not yet been reported (15). Notably, a subgroup analysis in PD-L1 CPS <1 TACTI−003/KEYNOTE−C34 trial suggested an OS benefit for the combination of efti and pembrolizumab in the first−line treatment of a small cohort of 31 patients with R/M HNSCC and PD−L1 CPS < 1 (23). To date, these results have not been published. Other multi−checkpoint inhibition strategies—including dual PD−L1/TIGIT blockade with atezolizumab plus tiragolumab in the SKYSCRAPER−09 trial, as well as the addition of anti−LAG−3 or combined anti−LAG−3 and anti−TIM−3 to anti−PD−1 therapy—failed to improve progression−free survival (PFS) compared with anti−PD−1 monotherapy in PD−L1–positive R/M HNSCC, despite evidence of augmented immunologic activity (16, 17).

An alternative therapeutic strategy has focused on modifying the tumor microenvironment through the combined inhibition of VEGF and PD−1. This approach aims to normalize tumor vasculature, reduce hypoxia, limit infiltration by regulatory T cells and myeloid−derived suppressor cells, and promote dendritic cell maturation (24). In line with this rationale, the combination of pembrolizumab and lenvatinib—a multikinase inhibitor targeting VEGFR1−3, FGFR1−4, PDGFRα, RET, and KIT—resulted in a significantly higher objective response rate compared with pembrolizumab alone (46.1% vs. 25.4%; p < 0.00001). However, this improvement did not translate into an OS benefit (HR 1.15; 95% CI 0.91–1.45; p = 0.882) (18). Similarly, macrophage−targeting strategies such as the combination of evorpacept, a CD47−blocking agent, with pembrolizumab +/-and chemotherapy in the ASPEN−03 and ASPEN04 trial, although biologically sound and associated with encouraging response signals, have thus far failed to demonstrate a definitive survival advantage over standard pembrolizumab−based regimens in the first−line R/M HNSCC setting (K.J. 19, 20).

## 5 Revisiting EGFR as a therapeutic axis: a strategic partnership

Within this therapeutic landscape, epidermal growth factor receptor (EGFR) signaling has re−emerged as a biologically relevant axis in the post–anti−PD−1 setting, reflecting its central role in HNSCC pathogenesis, particularly in HPV−negative disease. Monotherapy with cetuximab resulted in an ORR of 23.9% in the INTERLINK−1, while the addition of monalizumab (anti-NKG2A antibody) to cetuximab had no benefit (25). A Chinese retrospective study reported an ORR of 46% of combined cetuximab + pembrolizumab in anti-PD1 resistant HNSCC, with a mPFS and mOS of 6.9 and 9.7 months, respectively (26). Beyond immune checkpoint inhibition, the combination of paclitaxel and cetuximab has also demonstrated substantial clinical activity, achieving an objective response rate of 69% and median progression−free and overall survival of 5.5 and 13.3 months, respectively (27).

Despite its long−standing use, the clinical efficacy of cetuximab in HNSCC remains modest, with both primary and acquired resistance frequently observed. Mechanistic studies have identified multiple resistance pathways, including persistent downstream signaling through the MAPK and PI3K/AKT pathways, compensatory activation of parallel receptor tyrosine kinases such as HER2, HER3, and MET, and EGFR−independent oncogenic drivers (28). Additional factors, including altered EGFR internalization and recycling, ligand overexpression, and intratumoral heterogeneity, further limit the durability of responses (29). To address these limitations, a new generation of EGFR−directed agents is being developed that moves beyond classical ligand blockade, with the aim of overcoming both EGFR−mediated and anti−PD−1−associated resistance phenotypes.

**Figure 2:**
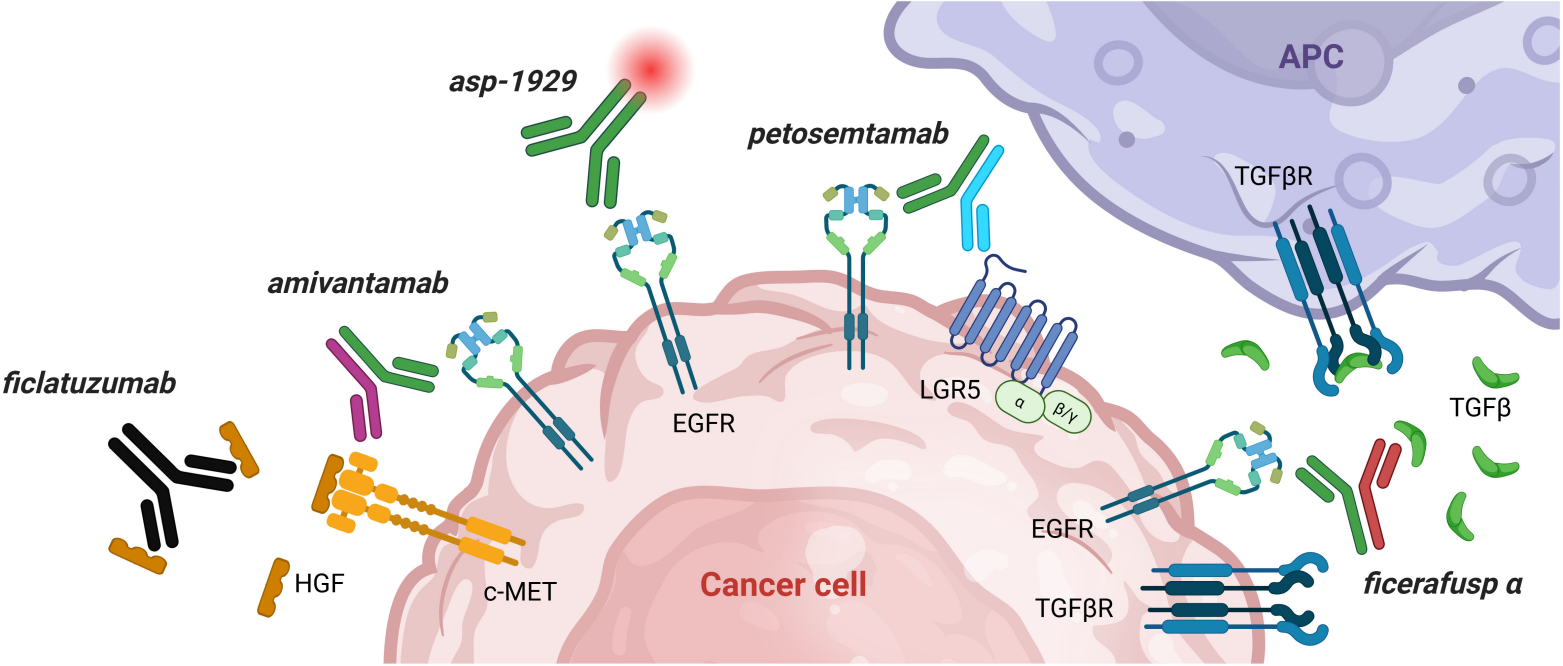
Overview of novel EGFR-targeting agents in HNSCC. EGFR is overexpressed in >80% of HNSCC. Traditional EGFR blockade with cetuximab often leads to primary and acquired resistance. Overcoming resistance can be done by co-targeting EGFR with known resistance pathways: (1) Bispecific antibodies co-targeting EGFR induce block ligand binding, induce receptor degradation, and stimulate immune-mediated antitumor activity (ADCC). *Amivantamab* is a bispecific antibody targeting EGFR and c-MET, a known resistance pathway to cetuximab. *Petosemtamab* is bispecific antibody targeting EGFR and the cancer stem cell marker LGR5. *Ficerafusp alfa* is a bispecific antibody targeting EGFR and TGFβ, reducing immune-inhibitory signals. (2) combination of *cetuximab* with *ficlatuzumab* (anti-HGF) results in selective inhibition of the HGF/c-MET pathway and has demonstrated potent suppression of downstream c−MET signaling. (3) *asp-1929: conjugated cetuximab conjugated to a light-activatable dye (IRDye 700DX)*: when binding of a tumor cell with cetuximab, cell becomes illuminated with red light (690nm), resulting in selective destruction of EGFR+ tumor cells. ADCC, antibody−dependent cellular cytotoxicity; APC, antigen presenting cell; EGFR, epidermal growth factor receptor; HGF, hepatocyte growth factor; LGR5, leucine-rich repeat-containing G-protein coupled receptor 5; R/M HNSCC, recurrent/metastatic head and neck squamous cell carcinoma; TGFβ, transforming growth factor β. Figure created in BioRender.

In this context, bispecific antibodies have entered clinical development and are currently under active investigation in the first−line treatment of R/M HNSCC following encouraging early−phase results (see **Table 2** and **Figure 2**).

**Table 2:** Novel anti-EGFR based treatments in R/M HNSCC.

Author, year	Intervention	Population (N)	ORR	mPFS	mOS
Fayette, 2025 (25)	Cetuximab	2-3L, after ICI and platinum, HPV neg (N=)	24%	3.8m	8.6m
	Cetuximab + monalizumab	2-3L, after ICI and platinum, HPV neg (N = 216)	15%	3.6m	8.8m
Hui, 2024 (26)	Pembrolizumab + cetuximab	2-3L, after ICI and platinum (N = 28)	46%	6.9m	9.7m
Koyama, 2023 (27)	Paclitaxel + cetuximab	2-3L, after ICI and platinum (N = 35)	69%	5.5m	13.3m
Harrington, 2025(Kevin J. 19)	Amivantamab	2-3L after ICI and platinum (N = 86)	45%	6.8m	NR
Le Tourneau 2024 (30)	Petosemtamab	2-3L after ICI and platinum (N = 110)	37%	4.9m	11.4m
Fayette, 2024 (31)	Petosemtamab + pembrolizumab	1L, PD-L1 CPS >1 (N = 26)	63%	NA	NA
Kaczmar, 2025 (32)	Ficerafusb alfa + pembrolizumab	1L, PD-L1 CPS >1 (N = 31)	57%	7.4m	NA
Bauman, 2023 (33)	Ficlatuzumab + cetuximab	2-3L, anti-PD1 and cetuximab resistant	38%	4.1m	7.4m
Cognetti, 2025 (34)	ASP-129 + pembrolizumab	1L, PD-L1 CPS >1 (N = 18)	28%	2.9m	25m

R/M HNSCC, relapsed or metastatic head and neck squamous cell carcinoma; PD-L1 CPS, combined positivity score of PD-L1 expression; ORR, objective response rate; mPFS, median progression-free survival; mOS, median overall survival; NA, not available; NR, not reached.

### 5.1 Amivantamab

Amivantamab is a bispecific antibody targeting both EGFR and MET and exerts its antitumor activity through a triple mechanism of action, combining direct EGFR inhibition, MET inhibition—an important escape pathway in the context of anti EGFR therapy—and immune cell directing activity. The clinical activity of amivantamab in HNSCC was explored in the OrigAMI 4 phase 1b/2 trial, which evaluated subcutaneous amivantamab in patients with HPV negative R/M HNSCC who had previously been treated with platinum based chemotherapy and PD-1 inhibitors, and who had not received prior anti EGFR therapy. Despite this heavily pretreated setting, amivantamab demonstrated substantial clinical activity, achieving an ORR of 45% with a median duration of response of 7.2 months. Tumor shrinkage was observed in the majority of treated patients, and the safety profile was consistent with the known tolerability of amivantamab, with predominantly infusion related reactions, skin toxicity, and paronychia (35).

Based these encouraging results, a phase III study (OrigAMI 5) is currently actively recruiting. This trial evaluates subcutaneous amivantamab in combination with pembrolizumab and carboplatin, compared with the standard pembrolizumab based chemotherapy regimens of pembrolizumab plus 5 fluorouracil and either cisplatin or carboplatin, as first line treatment for patients with R/M HNSCC (NCT07276399).

### 5.2 Petosemtamab

Petosemtamab is a human IgG1 bispecific antibody with a common light−chain format and enhanced antibody−dependent cellular cytotoxicity (ADCC), simultaneously targeting EGFR and the cancer stem cell marker LGR5. Its clinical activity has been evaluated across multiple treatment lines in R/M HNSCC. In a phase II study assessing petosemtamab monotherapy in the second− and third−line setting, treatment resulted in an ORR of 37.2%, with a median duration of response of six months (30). Building on this activity, updated data from a first−line phase II study evaluating petosemtamab in combination with pembrolizumab demonstrated an unprecedented ORR 63% among patients with PD−L1 CPS ≥ 1, with 12−month progression−free and OS rates of 38% and 78%, respectively. Importantly, responses were observed in both HPV−positive and HPV−negative disease, suggesting activity across biologically distinct HNSCC subtypes (31).

Petosemtamab was generally well tolerated, with the most frequently reported adverse events including acneiform dermatitis, asthenia, rash, and infusion−related reactions, consistent with on−target EGFR inhibition and immune engagement (30, 31). Further clinical development is ongoing, including a randomized phase III trial evaluating petosemtamab plus pembrolizumab as first−line therapy for R/M HNSCC (NCT06525220), as well as a comparative study of petosemtamab versus investigator’s choice in the second− and third−line settings (NCT06496178).

### 5.3 Ficerafusp alfa

Ficerafusp alfa is a bispecific fusion protein designed to simultaneously inhibit EGFR and neutralize transforming growth factor−β (TGF−β), a central mediator of immunosuppressive signaling within the HNSCC tumor microenvironment. By combining direct tumor signaling inhibition with modulation of the immune milieu, ficerafusp alfa represents an integrated approach to overcoming immune resistance. Early−phase clinical data evaluating ficerafusp alfa at a dose of 1500 mg in combination with pembrolizumab in first−line HPV−negative R/M HNSCC demonstrated substantial antitumor activity, with an ORR of 54%, including 21% achieving a complete response. Responses occurred fast and durable, with a median time to response of 1.4 months, a median duration of response of 21.7 months (32).

The safety profile of ficerafusp alfa was largely characterized by dermatologic and constitutional adverse events, most commonly acneiform dermatitis, pruritus, and fatigue, consistent with EGFR−related toxicity patterns. Ongoing confirmatory evaluation is underway in the FORTIFI−HN01 trial, a global, multicenter, randomized, double−blind phase II/III study comparing ficerafusp alfa versus placebo in combination with pembrolizumab in patients with HPV−negative, PD−L1−positive R/M HNSCC (NCT06788990).

### 5.4 Ficlatuzumab plus cetuximab

Ficlatuzumab, a monoclonal antibody directed against HGF, provides selective inhibition of the HGF/c-MET pathway and has demonstrated potent suppression of downstream c−MET signaling, with additive antitumor effects in both preclinical models and early−phase clinical studies.

A randomized phase II trial assessed the clinical activity of ficlatuzumab, with or without cetuximab, in patients with R/M HNSCC progressing on >2 lines of treatments including anti-PD1. The most favorable outcomes were observed in the HPV−negative subgroup treated with the combination, which achieved a median PFS of 4.1 months and an ORR of 38% (33). These results support the biological rationale for simultaneous EGFR and HGF/c−MET pathway inhibition in this molecularly defined population. A randomized phase 3 trial is ongoing, evaluating ficlatuzumab in combination with cetuximab in patients with HPV−negative R/M HNSCC post anti-PD1 (NCT06064877).

### 5.5 ASP-1929

ASP-1929 photoimmunotherapy is a drug–device combination, in which the EGFR-targeting agent cetuximab is conjugated to a light-activatable dye, IRDye 700DX. Cells expressing EGFR are bound with the antibody conjugate and subsequently illuminated with red light (690 nm) for localized activation of the dye, resulting in selective destruction of tumor cells while sparing surrounding tissues. In a small phase Ib/II trial, ASP-1929+pembrolizumab resulted in a significant clinical activity (ORR 27.8%, mOS 25 months). The addition of ASP-1929 resulted in increased local symptoms including oral pain, tong swelling and laryngeal edema (34). A phase III randomized study is currently enrolling patients to evaluate ASP−1929 photoimmunotherapy in combination with pembrolizumab versus standard−of−care treatment in locoregionally recurrent HNSCC (NCT06699212).

Collectively, these therapeutic strategies exemplify integrative approaches that simultaneously target tumor−intrinsic signaling and microenvironmental immune resistance, addressing the multifaceted nature of treatment failure in R/M HNSCC. An overview of the ongoing clinical trials with novel EGFR inbitors is provided in **Table 3**.

**Table 3:** Overview of recruiting phase 3 trials in first line treatment of R/M HNSCC (accessed via clinicaltrials.gov on Jan 15 2026).

NCT	Trial name	Intervention (arm)	Control (arm)	Population (key)	Study completion
Local treatment					
NCT05815927	EORTC-2014-HNCG	Pembrolizumab + radiotherapy	pembrolizumab	Oligometastatic HNSCC, PD-L1 ≥1	2030
NCT04747054	PembroMetaRT	Pembrolizumab + radiotherapy	Pembrolizumab maintenance	M+ HNSCC, PD-L1 ≥1	2029
NCT05721755		Pembrolizumab + radiotherapy	Pembrolizumab maintenance	M+ HNSCC, PD-L1 ≥1	2030
Systemic treatment: all comers					
NCT07264075	ILLUMINE	Ivonescimab +/- ligufalimab	pembrolizumab	R/M HNSCC, PD-L1 ≥1	2030
NCT06295731	HexAgon-HN	INBRX-106 + pembrolizumab	pembrolizumab	R/M HNSCC, PD-L1 ≥1	2029
NCT06601335		AK112+AK117	pembrolizumab	R/M HNSCC, PD-L1 ≥1	2027
NCT06525220	LiGeR-HN1	Petosemtamab + pembrolizumab	pembrolizumab	R/M HNSCC, PD−L1 ≥1	2030
NCT06788990	FORTIFI−HN01	Ficerafusp alfa + pembrolizumab vs	placebo + pembrolizumab	R/M HNSCC, PD−L1 ≥1, HPV neg	2029
NCT07276399	OrigAMI-5	Amivantamab + carboplatin + pembrolizumab	Pembrolizumab + carboplatin + 5FU	R/M HNSCC, all PD-L1	2029
NCT06589804		Pembrolizumab + cetuximab	Pembrolizumab	R/M HNSCC, PD-L1 ≥1, HPV neg	2029
NCT06769698		Fianlimab + cemiplimab	Cemiplimab	R/M HNSCC, PD-L1 ≥1	2030
NCT05913388		GB1211 + pembrolizumab	pembrolizumab	R/M HNSCC, all PD-L1	2030
Systemic treatment: 1st line HPV+					
NCT06790966	VERSATILE−003	PDS0101 + pembrolizumab	pembrolizumab	1L HPV16+ R/M HNSCC	2029
NCT04534205	AHEAD-MERIT	BNT-113 + Pembrolizumab	Pembrolizumab	R/M HNSCC, PD-L1 ≥1, HPV+	2029

## 6 HPV−directed therapeutic vaccines: reprogramming antitumoral immunity

HPV16 accounts for the vast majority of oropharyngeal cancers and is associated with a clearly distinct tumor biology compared with HPV−negative disease (36, 37). Persistent expression of the viral oncoproteins E6 and E7 shapes a markedly inflamed tumor microenvironment, characterized by increased infiltration of CD4+ and CD8+ T cells, enrichment of antigen−presenting cells, and elevated levels of pro−inflammatory cytokines and chemokines (38, 39).

Despite this viral etiology and immunologically “hot” phenotype, HPV−positive HNSCC has not demonstrated the degree of sensitivity to PD−1 blockade that was initially anticipated in clinical trials (13, 40, 41). Chronic exposure to viral antigens drives functional exhaustion of tumor−infiltrating T cells, reflected by high expression of inhibitory receptors including PD−1, LAG−3, TIGIT, and TIM−3 (42). In addition, specific HPV oncoproteins, such as E5, can directly disrupt antigen processing and presentation, thereby promoting immune evasion and contributing to resistance to PD−1/PD−L1 blockade in preclinical models (43).

**Figure 3:**
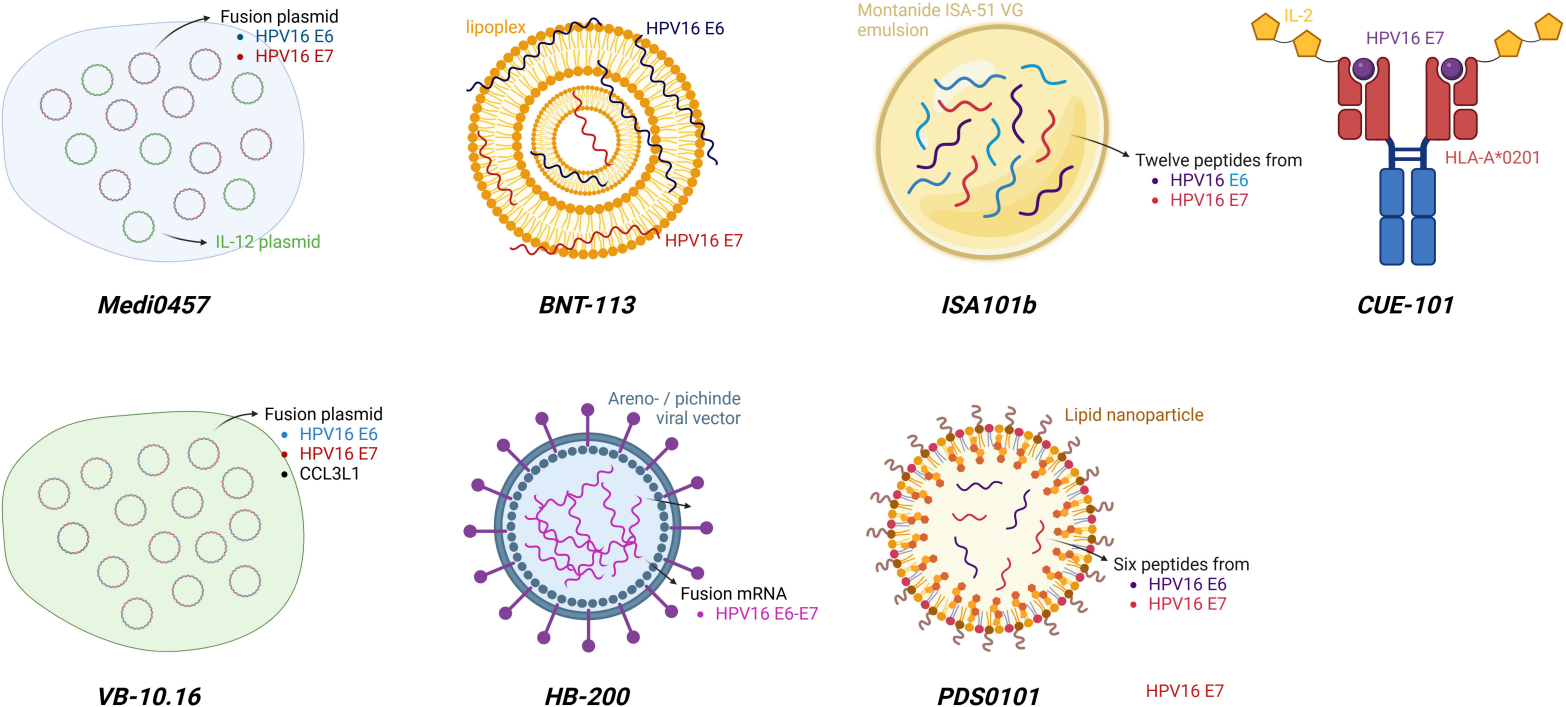
Overview of HPV16 directed therapeutic vaccines currently studied in HNSCC. Therapeutic vaccines to expand and reinvigorate HPV16−specific T cells, enhance antigen presentation, and reshape the tumor microenvironment toward a more permissive immune state. Current approaches can be distinguished in four different platforms: (1) DNA plasmid vaccines (*Medi0457* and *VB-10.16)*: After intramuscular administration and plasmid transfection, endogenous expression of HPV16 E6 and E7 antigens triggers HPV-specific immune responses. Immune activation is further potentiated by co-expression of IL-12 (*Medi0457*) or CCL3L1 (*VB-10.16*). (2) mRNA vaccines: HPV16 E6 and E7 mRNA transcripts are embedded in a lipoplex (*BNT-113*) or an arenovirus vector (*HB-200*) and administered intravenously, to induce E6 and E7-specific T cell responses. (3) Peptide vaccines: HPV16 E6 and E7 peptides coated in a specific emulsion (*ISA101b*) or in a lipid nanoparticle (*PDS0101*) are administered subcutaneously, and can elicit a specific antitumor response after recognition by APC. (4) Fusion proteins: *CUE-101* vaccine was developed as a Fc fusion protein composed of a human leukocyte antigen complex, an HPV16 E7 peptide epitope, and reduced affinity human IL-2 molecules. As such, this molecule can mimic the function of an APC and directly active T-cells. APC, antigen presenting cell; CCL3L1, Chemokine-ligand-3-like 1; HPV, human papillomavirus; IL, interleukin; HNSCC, head and neck squamous cell carcinoma. Figure created in BioRender.

Consequently, overcoming resistance to anti−PD−1 therapy in HPV−positive HNSCC is likely to require therapeutic strategies that actively reprogram dysfunctional antiviral T−cell responses and restore effective tumor−specific immunity. Such approaches aim to expand and reinvigorate HPV−specific T cells, enhance antigen presentation, and reshape the tumor microenvironment toward a more permissive immune state. These strategies are implemented across multiple vaccine platforms—ranging from peptide-based and nucleic acid–based constructs to viral vector systems—which differ in delivery mechanisms but converge on the induction of robust and durable remissions. Several of these therapeutic vaccines have already demonstrated encouraging early clinical activity in patients with HPV−positive R/M HNSCC, especially in anti-PD1 naïve patients (see **Figure 3** and **Table 4**).

**Table 4:** Early phase clinical data from therapeutic vaccines in HPV16+ R/M HNSCC.

Author, year	Intervention	Mechanism	Population (N)	ORR	mPFS	mOS
Weiss, 2025 (44)	PDS0101 + pembrolizumab	peptide	1L R/M HPV+, PD-L1 CPS >1	36%	NA	30m
Colevas, 2024 (45)	Cue-101 + pembrolizumab	Fusion protein	1L R/M HPV+, PD-L1 CPS >1	46%	5.8m	NR
Colevas, 2024 (45)	Cue-101	Fusion protein	2L R/M HPV+, anti-PD1 resistant	5%	NA	21m
Ho, 2024 (46)	HB-200 + pembrolizumab	Arenavirus-based	1L R/M HPV+, PD-L1 CPS >1	37%	16.3m	NR
Saba, 2024 (47)	BNT-113 + pembrolizumab	mRNA	1L R/M HPV+, PD-L1 CPS >1	40%	NA	NA
Even, 2024 (48)	ISA101b + cemiplimab	peptide	1L/2L R/M HPV+, PD-L1 CPS >1, antiPD1 naïve	26%	NA	NA
Kong, 2024 (49)	Isa101b + cemiplimab	peptide	2L R/M HPV+, anti-PD1 resistant	11.5%	3.6m	11m
Aggarwal, 2023 (50)	Medi0457 + durvalumab	peptide	1L R/M HPV+, PD-L1 CPS >1	28%	3.5m	29m

HPV16, human papillomavirus serotype 16; R/M HNSCC, relapsed or metastatic head and neck squamous cell carcinoma; PD-L1 CPS, combined positivity score of PD-L1 expression; ORR, objective response rate; mPFS, median progression-free survival; mOS, median overall survival; NA, not available; NR, not reached.

### 6.1 PDS0101

PDS0101 is a lipid nanoparticle vaccine injected subcutaneously, delivering multiple HPV16 peptides. In the phase II VERSATILE-002 study, PDS0101 combined with pembrolizumab showed encouraging antitumor activity with an ORR of 36% and mOS of 30 months, prompting a phase III randomized study (NCT06790966) (44).

### 6.2 CUE-101

CUE-101 is a fusion protein composed of a human leukocyte antigen (HLA) complex derived from HPV16 E7 protein and a complex of attenuated human interleukin-2 (IL-2) molecules, which mimics antigen presenting cells via targeted cytokine delivery and leads to a selective expansion of antigen-specific T cells. The combination of CUE-101 and pembrolizumab in first line R/M HPV+ HNSCC resulted in an had a meaningful activity (ORR of 46%, mPFS of 5.8m., mOS NR), whereas second line activity was limited (ORR 5%). Up to 30% of patients experienced infusion reactions and transient fever in the first injections. (45) Further evaluation of CUE-101 is ongoing in the neoadjuvant setting, in combination with pembrolizumab (NCT07172256).

### 6.3 HB-200

The arenavirus-based viral vector vaccine HB-200, composed derived from the lymphocytic choriomeningitis (HB-201) and Pichinde viruses (HB-202). It expresses an HPV-16 E7E6 fusion protein and as monotherapy has been shown to induce HPV-16 tumor-specific T-cell responses in heavily pretreated patients (51). In combination with pembrolizumab, HB-200 resulted in an ORR of 37% in first-line HPV+ R/M HNSCC (46). About 30% of patients experienced transient pyrexia/influenza like illness after injection. Further development has been paused due to restructuring plan at the company (52).

### 6.4 BNT-113

BNT113 is an investigational uridine-based mRNA-lipoplex (LPX) cancer immunotherapy encoding the HPV-16 oncoproteins E6 and E7, leading to DC maturation and induction of profound antigen-specific T cell responses. The first interim evaluation (N = 15) of the combination of BNT-113 with pembrolizumab in first-line HPV-driven R/M HNSCC yielded an ORR of 40%. Survival data are maturing. The combination appeared safe with mainly influenza-like illness and fever as adverse events (47) Currently, a randomized phase II/III trial is recruiting (NCT04534205).

### 6.5 DNA plasmid vaccines: abipapogene suvaplasmid VB-10.16 (abi-suva)

Abi-suva is a potent DNA plasmid vaccine with intrinsic adjuvant effect designed for efficient delivery of antigens E6 and E7 from HPV16 to elicit strong immune responses. It has been evaluated in combination with atezolizumab in pretreated cervical cancer patients, showing an ORR of 29% in PD-L1+ patients, without increasing side effects. (53) It is currently under investigation in a randomized phase I/II trial in combination with pembrolizumab in 1st line R/M HNSCC (NCT06016920).

### 6.6 ISA101b

ISA101b is a peptide vaccine encoding for the oncoproteins E6 and E7 that has been evaluated in a randomized trial combination with cemiplimab vs cemiplimab monotherapy. While the primary endpoint of ORR was not met in all-comers, the subgroup with PD-L1 CPS >20 exhibited an increased ORR (51.7 vs 28.8%) and mOS (34.8 vs 28.8 months) (48). In anti-PD1 resistant patients, the activity was limited (ORR 11.5%, mOS 8 months) (49). To date, it is unclear whether ISA101b will be further developed in a phase 3 trial.

### 6.7 Medi0457

Medi0457 is a peptide vaccine targeting HPV-16/18 E6/E7 with an IL12 adjuvant. Combination with durvalumab was evaluated in a phase I/II trial in 35 platinum-pretreated ICI-naïve patients. The study missed the primary efficacy endpoint (ORR 27.6%), and further development has been halted (50).

These data indicate that multiple HPV-directed agents yielded promising results in early phase trials, mostly in high PD-L1 expressing tumors and ICI-naïve patients. Ongoing phase III studies (see **Table 3**) will determine whether vaccine−anti−PD−1 combinations can provide an additional benefit over the current standards.

## 7 Cellular, viral and targeted modalities in HNSCC: expanding the portfolio

### 7.1 Adoptive cellular therapy

Transferring autologous tumor−infiltrating lymphocytes (TILs) has also emerged as a promising immune−based strategy in ICI-refractory tumors such as melanoma (54). Lifileucel (LN-144) is an autologous TIL therapy that uses tumor-tissue T cells capable of recognizing tumor antigens and being expanded ex vivo while maintaining the heterogeneous repertoire of T cells, using a centralized manufacturing process. In a recently published phase II study, a single administration of lifileucel achieved a disease control rate of 76% in patients with R/M HNSCC, including individuals whose disease had progressed despite multiple prior lines of therapy. The median duration of response was 7.6 months, while the median overall survival reached 9.5 months (55). Adoptive cellular therapy is an intensive treatment requiring myeloablative chemotherapy and prolonged hospitalization. The most common grade 3/4 treatment-emergent AEs were febrile neutropenia (42%) and hypotension (23%), apart from transient hematotoxicity. Notably, three grade 5 TEAEs occurred, due to respiratory failure (55). Identification of patients who may be candidates for TIL cell therapy is challenging, and the treatment window in which patients may be eligible could be narrow.

### 7.2 Oncolytic and therapeutic vaccines

Similar to HPV+ disease, oncolytic viral therapies aim to augment tumor-specific reactive T cells in HPV- disease. The main challenge is finding and manufacturing host-specific neoantigens to prime the immune response, which may be overcome by personalized cancer vaccines. TG4050 is a viral-based personalized cancer vaccine, encoding up to 30 tumor-specific DNA sequences bearing in-silico predicted class I and class II epitopes. In a randomized phase I study, TG4050 reduced recurrences in LA HNSCC undergoing surgery (56). A randomized phase 3 study evaluating TG4050 in the adjuvant phase of LA HNSCC is ongoing (NCT06699212).

### 7.3 Antibody-drug conjugates

Antibody–drug conjugates (ADCs) represent a rapidly expanding therapeutic class that is increasingly reshaping the field of oncology, composed of a specific antibody linked to a cytotoxic (anticancer) “payload” or drug. (57, 58). ADCs may be administered either sequentially or in combination with immune checkpoint inhibitors, with the aim of enhancing antitumor activity and potentially re−sensitizing tumors to immunotherapy (59). In this regard, both enfortumab vedotin (targeting Nectin-4) and brentuximab vedotin (targeting CD30) in combination with pembrolizumab have demonstrated encouraging clinical activity in treatment-naïve R/M HNSCC patients with PD-L1 CPS >1, with an ORR of 39% and 34%, respectively (60, 61). Other ADC+ICI combinations are under evaluation in early phase clinical trials (NCT07088211, NCT03485209). Encouraging activity of ADCs was also seen in anti-PD1 refractory patients with tizotumab vedotin (targeting tissue factor), ozuriftamab vedotin (targeting ROR) and sigvotagug vedotin (targeting integrin-B6) with ORR of 32, 36 and 23%, respectively. The toxicity management of ADCs can be challenging, particularly in heavily pretreated patients with HNSCC who often experience rapid deterioration in performance status. For example, tisotumab vedotin was associated with severe (grade ≥3) peripheral neuropathy in 12.5% of patients; ocular (52%) and bleeding events (37%) were also frequently observed. Given the number of ADC platforms, targets, and combinatorial approaches currently under clinical evaluation, the ADC landscape in HNSCC remains highly dynamic and is likely to undergo substantial refinement over the coming years.

### 7.4 Radioligand therapy

Radioligand therapy (RLT) has emerged as an active and rapidly evolving area of research within oncology and has already secured a place in routine clinical practice for tumor types such as neuroendocrine malignancies and prostate cancer. (62, 63) In contrast, experience with RLT in HNSCC remains limited and remains at a formative stage (64). To date, diagnostic applications have been explored using radionuclides such as 89Zr and 64Cu conjugated to EGFR−targeting monoclonal antibodies, enabling high−resolution imaging of tracer uptake in patients with HNSCC. (65, 66) The development of therapeutic radiopharmaceuticals for HNSCC, however, has lagged behind, despite the overexpression of appealing suitable anchors for localized delivery of targeted ionizing radiation, such as EGFR and HER3. Key challenges include the a more comprehensive understanding of treatment mechanisms, optimization of dosing and treatment protocols, and the development of radiopharmaceuticals specifically tailored to the biological characteristics of HNSCC.

### 7.5 Local treatment in the immunotherapy area

Local treatment of the primary tumor in patients with metastatic disease is a reasonable approach, that gained increased traction in the era of immunotherapy (67) This presumption has already been confirmed in nasopharyngeal cancer in which the addition of definitive locoregional radiotherapy to systemic treatment in metastatic patients improved their overall survival (68). In R/M HNSCC, a retrospective analysis of the National Cancer Data Base including 3269 patients found that an aggressive local approach to the primary tumor (radiotherapy ≥ 60 Gy or oncologic resection) as an adjunct to palliative systemic treatment improved three-year OS (34.2% versus 20.6%, p <.001). A benefit was observed in all evaluated subgroups categorized according to age, sex, T-stage, N-stage, anatomic site, type of systemic therapy, comorbidities, academic versus non-academic centers, facility volume, insurance status, income, education, and urban versus non-urban areas (69). Another retrospective study including 40 HNSCC patients with synchronous distant metastases that received definitive treatment to the primary site had favorable outcomes with mPFS and mOS of 11.3 months and 41.7 months, respectively, for those who received a lead-in with an ICI-based regimen (70). While the approach is rational, the evidence currently is only based on retrospective studies. Currently, three prospective trials are investigating the addition of local treatment in recurrent and/or (oligo)metastatic HNSCC (see **Table 3**).

## 8 Translational considerations: matching the right drug to the right patient

The major challenge in the coming decade is unlikely to be the absence of therapeutic options, but rather the absence of validated strategies to match individual patients to the most effective treatment. Indeed, the identification of robust biomarkers capable of predicting clinical benefit from these novel, innovative treatments will be critical to enable precise patient stratification, optimize treatment sequencing, minimize unnecessary toxicity, and ultimately improve survival and quality of life for patients with relapsed or metastatic HNSCC. If not, the field risks entering an era of empiric combinatorial escalation with increasing toxicity and cost. A recent study showed that among 29 registrational trials in breast cancer, none resulted in biomarker research restricting the drug indication (71). Notably, translational research data from the pivotal Keynote-048 trial are still unpublished, despite the trial results were first reported in 2018. Biospecimens collected in clinical trials can provide valuable information on both efficacy and resistance pathways, essential for effective patient stratification and treatment sequencing. Addressing these knowledge gaps will require a strategic partnership between (academic) translational researchers and pharmaceutical companies, ensuring access to the collected samples to facilitate biomarker discovery and enable mechanistic insight into efficacy, resistance and toxicity, and guide the rational development and sequencing of next−generation therapies in R/M HNSCC.

## 9 Conclusion and roadmap

Where ICIs were a major breakthrough in R/M HNSCC, further improvements have been absent since their approval. Several next-generation EGFR-directed bispecifics and immune-modulating fusion proteins have generated sufficiently robust early-phase signals that provide hope for meaningful improvements in patient outcomes and the evolution of personalized treatment strategies. In the coming decade, progress in R/M HNSCC will depend less on the number of available agents than on our ability to define biologically meaningful disease subsets and assign the right therapy to the right patient. Based on the available phase I/II clinical evidence, bispecific antibodies such as amivantamab, petosemtamab, and ficerafusp alfa are emerging as promising therapeutic options that may, in the near future, be incorporated either as novel first−line treatment strategies or as effective interventions following progression on anti−PD−1–based and platinum−containing regimens. In parallel, combinations of therapeutic vaccination with PD−1 blockade have demonstrated their greatest promise in HPV16−positive disease and may substantially reshape first−line treatment paradigms should ongoing phase III trials confirm meaningful survival benefits. Moreover, optimal care will require a multidisciplinary approach, including consideration of local ablative strategies in (oligo)metastatic disease, and conversely a tailored systemic approach for widespread disease. Until these definitive data become available, enrollment in clinical trials remains a key priority for eligible patients with HNSCC.
